# Beauty and Wellness in the Semantic Memory of the Beholder

**DOI:** 10.3389/fpsyg.2021.696507

**Published:** 2021-08-05

**Authors:** Yoed N. Kenett, Lyle Ungar, Anjan Chatterjee

**Affiliations:** ^1^Penn Center for Neuroaesthetics, University of Pennsylvania, Philadelphia, PA, United States; ^2^Faculty of Industrial Engineering & Management, Technion–Israel Institute of Technology, Haifa, Israel; ^3^Department of Computer and Information Science, University of Pennsylvania, Philadelphia, PA, United States

**Keywords:** beauty, wellness, semantic networks, word2vec, aging

## Abstract

Beauty and wellness are terms used often in common parlance, however their meaning and relation to each other is unclear. To probe their meaning, we applied network science methods to estimate and compare the semantic networks associated with beauty and wellness in different age generation cohorts (Generation Z, Millennials, Generation X, and Baby Boomers) and in women and men. These mappings were achieved by estimating group-based semantic networks from free association responses to a list of 47 words, either related to *Beauty*, *Wellness*, or *Beauty* + *Wellness*. *Beauty* was consistently related to *Elegance*, *Feminine*, *Gorgeous*, *Lovely*, *Sexy*, and *Stylish*. *Wellness* was consistently related *Aerobics*, *Fitness*, *Health*, *Holistic*, *Lifestyle*, *Medical*, *Nutrition*, and *Thrive*. In addition, older cohorts had semantic networks that were less connected and more segregated from each other. Finally, we found that women compared to men had more segregated and organized concepts of *Beauty* and *Wellness*. In contemporary societies that are pre-occupied by the pursuit of beauty and a healthy lifestyle, our findings shed novel light on how people think about beauty and wellness and how they are related across different age generations and by sex.

## Introduction

Beauty seems to be a pervasive human pre-occupation (Grammer et al., 2003; Zangwill, 2005, 2018; Scruton, 2011, 2018; Sartwell, 2017). We aspire to be beautiful, surround ourselves with beautiful things, and create beautiful artifacts (Bloch and Richins, 1992; Chatterjee, 2014; Redies, 2014). Beauty is attributed to natural objects (e.g., faces landscapes, animals, etc.) as well as to cultural artifacts (e.g., art and architectural design; Menninghaus et al., 2019). Despite this pre-occupation with beauty, we have little clarity on what beauty means, and how it is linked to different kinds of valuation.

Examining this elusive concept based on its associations could provide some sense of how people conceptualize beauty (Chatterjee, 2014; Chatterjee and Vartanian, 2014; Menninghaus et al., 2019). According to the aesthetic triad framework (Chatterjee and Vartanian, 2014, 2016), an aesthetic experience, such as beauty, emerges from three components: A sensory-motor component, an emotional-evaluative component, and a meaning-knowledge component. Cognitive theory and research have linked the meaning of concepts to their semantic neighborhoods, e.g., concepts that are directly linked to it (Klimesch, 1987; Landauer and Dumais, 1997; Günther et al., 2019). Thus, a conceptual space of beauty may be inferred from the concepts to which it is linked.

In the current study, we focus on the meaning-knowledge component of the aesthetic triad, by analyzing the natural language of participants. Beauty is also linked to other kinds of valuation, such as judgments of good (Dion et al., 1972; Eagly et al., 1991), and ideas of wellness (Koskinen et al., 2017). In this study, we also query the concept of wellness and its relation to beauty. We apply computational network science methods to natural language to determine how people’s concepts of beauty and wellness are similar or vary by age cohorts and by sex.

Evolutionary psychology research highlights notions of beauty as reflecting a preference for attractive people over unattractive people when choosing mates (Grammer et al., 2003). Valuation of beauty extend into other kinds of valuation, such as in judgments of morality, an extension often referred to as the “Beauty is good” (Dion et al., 1972; Eagly et al., 1991) stereotype. According to this stereotype, attractive people are judged to have positive personal characteristics; people who look good also are good. In a complementary line of research, faces with anomalies, such as scars, are rated by participants as having negative characteristics compared to non-anomalous faces (Hartung et al., 2019; Workman et al., 2020). This research suggests that our valuation systems are imprecise. In addition to conflating beauty and good, valuations of beauty also extend to wellness. Consistent with claims from evolutionary psychology, attractiveness signals mates who are healthy, to assure healthy off-springs (Grammer et al., 2003). Thus, the link between beauty and wellness may have deep evolutionary roots.

Concerns for wellness goes back many years. For example, Aristotle discussed how wellness is related to hedonia (the search for pleasure) and to eudaimonia (the cultivation of virtue; Huta and Waterman, 2014). In contemporary materially developed societies (Chen et al., 2015; Koskinen et al., 2017), wellness often refers to holistic health that emphasizes healthy self-interest, self-awareness, and self-improvement (Mueller and Kaufmann, 2001). Seeking physical and mental health is part of a meaningful lifestyle (Chen et al., 2015; Valentine, 2016; Lee et al., 2019). However, like beauty, wellness is challenging to define, and its meaning is malleable (Corbin and Pangrazi, 2001; Koskinen et al., 2017). Beyond health and medicine, wellness is associated with proactive practices such as meditation and exercise (Chen et al., 2015), a focus on self-realization, and the pursuit of pleasure (Ryan and Deci, 2001; Diener and Biswas-Diener, 2011; Ryff, 2018). This cultural trend toward wellness is reflected in a booming wellness industry (Lee et al., 2019), valued globally at 4.2 trillion dollars in 2017 (Global Wellness Institute, 2017). Thus, wellness is also a target of consumption (Featherstone, 2010). Given the range of associations of wellness to virtue, health, and leisure (Koskinen et al., 2017), how do people think about this concept as expressed in natural language?

The beauty industry often markets its products as reflecting health and inner beauty, suggesting that they capitalize on a psychological link between the two concepts. The logic of this marketing strategy, as mentioned above, receives support from evolutionary psychology. Sexual selection theories propose that an attractive, beautiful face and body signal healthy and fertile potential mates, with the most attractive individual expected to be the healthiest and most fertile (Buss and Schmitt, 1993; Grammer et al., 2003; Hönekopp et al., 2007; Coetzee et al., 2009; Stephen and Tan, 2015). Under these theories, beauty and wellness are both connected to physical health and reproductive success. This relationship may still be linked in our brains, even though the original factors that signaled health (e.g., immunocompetence, or susceptibility to parasites) and reproductive success, may be not be as relevant in the 21st Century (Chatterjee, 2014).

Given the potential overlap in how the concepts beauty and wellness relate to physical health, and the malleability of their meaning, how do they vary by age and sex? As people age they accumulate more experience and thus the knowledge component of these constructs may continue to develop (Park et al., 2002; Chatterjee and Vartanian, 2014). However, we know little about the effects of aging on the perception of beauty and wellness. For example, Koskinen et al. (2017) interviewed middle aged people (aged 50–65 years) on the topic of wellness. The authors found that wellness related activities were not aimed at directly combating ill-effects of aging, but were focused on sustaining a happy and fulfilled dally life (Koskinen et al., 2017).

To study how the conceptual space of thoughts about beauty and wellness vary by age, we need either longitudinal or cross-sectional research (Wulff et al., 2019). One approach is cross-sectional research on groups that differ in age and relate to different generations (Costanza et al., 2012, 2017; Rudolph et al., 2020a). While age cohorts refer to groups of individuals who are pooled together based on shared year of birth or specific significant events, age can also reflect variation between individuals associated with aging caused by physical and cultural maturation (Costanza et al., 2012).

To examine potential differences in the representation of these concepts across different age groups, we adopted an age cohort approach. Specifically, we targeted four cohorts, as defined by the Pew Research Center (Dimock, 2019): Generation Z (people born 1997–2012; 9–24 year old); Millennials (people born 1981–1996; 25–40 years old); Generation X (people born 1965–1980; 41–56 year old); and Baby Boomers (people born 1946–1964; 57–75 year old). Although individuals vary across generations, these generations are considered internally similar and distinct from each other (Costanza and Finkelstein, 2015; Rickes, 2016; Rudolph et al., 2020b). While individuals can certainly differ from their assigned group generalizations, typically Baby Boomers are described as idealistic optimists and educated; Generation X as cynical, disconnected, and practical skeptics; Millennials as realists, sheltered, and technological-savvy; and Generation Z as adaptive and social-media savvy (Rickes, 2016).

Finally, any difference in the meaning of beauty and wellness may also be related to sex differences, i.e., how men and women comprehend these constructs, and their relation, differently. For example, women rate physical beauty as less important than men do when ranking important attributes in attraction. The beauty market is also reshaping gender stereotypes and how men relate to beauty products. Contemporary men are more concerned with their own physical appearance and are more open to using cosmetics than were men who adopted traditional male gender stereotypes (Ostapenko, 2015; Khan et al., 2017). Such cultural changes raise questions about sex differences in the current perception of beauty and wellness. Taken together, the constructs of beauty and wellness are not well understood, and their meaning may vary by age and sex. We query the associations of beauty and wellness, their relationship, and by analyzing natural language examine how age and sex influence these concepts.

Our strategy of using natural language to examine beauty and wellness is motivated by the hypothesis that knowledge plays a role in aesthetic experiences (Chatterjee and Vartanian, 2014, 2016). Computationally analyzing verbal responses can measure, differentiate, and describe subjective experiences of psychological constructs (John et al., 1988; Kjell et al., 2019; Coburn et al., 2020; Hayn-Leichsenring et al., 2020). Several studies have used lexical corpora to analyze the semantics of aesthetics (Kuehnast et al., 2014; Hosoya et al., 2017; Menninghaus et al., 2019). Lexical corpus analysis—identifying the “semantic spaces” of concepts—is an indirect way of revealing associations of concepts, by representing conceptual spaces that make up such constructs (Günther et al., 2019). The “semantic neighborhood” of a concept can shed light on its meaning, even when the concept is hard to define directly. Kuehnast et al. (2014) analyzed free-association responses to emotional terms related to the concept of being-moved. The authors identified joy and sadness as key emotions related to the experience of being-moved, and showed how mapping the semantic space of being-moved, provided insight into components of this space (Kuehnast et al., 2014). In a recent study, Menninghaus et al. (2019) investigated how *Beauty* related to three similar aesthetics concepts: elegance, grace, and sexiness. Using free associations, questionnaires and semantic differential ratings (Menninghaus et al., 2019), they showed that elegance and grace differ from sexiness, but that all three concepts overlap in the broader category of beauty (Menninghaus et al., 2019). Their report is an example of the growing interest in representing the semantic space of aesthetic concepts such as beauty.

An approach to estimate semantic spaces from linguistic data is network science (Siew et al., 2019). Network science provides quantitative methods to investigate complex systems as networks (Newman, 2010; Barabási, 2016). A network is comprised of nodes, that represent the basic unit of the system (e.g., concepts in semantic memory) and links, or edges, that signify the relations between them (e.g., semantic similarity). Over the past two decades, several studies have used computational science to represent and study cognitive systems as networks (Borge-Holthoefer and Arenas, 2010; Baronchelli et al., 2013; Karuza et al., 2016; Siew et al., 2019). Specifically, such methods have been applied to investigate semantic memory—the cognitive system that stores facts and knowledge—as semantic networks (Siew et al., 2019; Kumar, 2021). For example, network science has tested psychological hypotheses that highly creative individuals have a more flexible semantic memory structure (Kenett et al., 2018; Kenett and Faust, 2019), identified mechanisms of language development (Steyvers and Tenenbaum, 2005; Hills et al., 2009), shed light on statistical learning (Karuza et al., 2017), shown how specific linguistic network properties influences memory retrieval (Vitevitch et al., 2012, 2014), and provided insight into the structure of semantic memory of second language in bilinguals (Borodkin et al., 2016).

In addition to these studies directed at cognition, network science can examine the effect of age cohorts on semantic memory (Zortea et al., 2014; Dubossarsky et al., 2017; Wulff et al., 2018, 2019; Cosgrove et al., 2021). These studies find that concepts in older adults’ semantic memory are more organized (i.e., concepts have sparser semantic neighborhoods, which means that concepts in the network are less connected) and more segregated (any pair of concepts in the network is “further apart”) than those of younger adults. For example, Dubossarsky et al. (2017) analyzed the network structure of free associations obtained from a cross-sectional cohort sample to estimate semantic networks for groups of young, middle-aged, and older adults. The authors found a U-shape change in semantic memory properties across the lifespan: Semantic memory starts off sparse, increases in density toward midlife, followed by increasing sparseness toward older ages (Dubossarsky et al., 2017).

We applied a semantic network approach to examine the following questions: (1) What are the semantic neighborhoods of the concepts *Beauty* and *Wellness*? (2) How are concepts of *Beauty* and *Wellness* linked to each other? (3) Do the concepts of *Beauty* and *Wellness* change across different age generations? And, as an exploratory question. (4) Do the concepts of *Beauty* and *Wellness* differ between men and women?

To address these questions, we conducted an online study in which we collected free-association responses to cue words related to *Beauty*, *Wellness*, and *Beauty* + *Wellness* (described later) from people of four age cohorts (Generation-Z, Millennials, Generation-X, and Baby Boomers). As mentioned earlier, free associations have been used to empirically study aesthetic concepts (Kuehnast et al., 2014; Hosoya et al., 2017; Menninghaus et al., 2019). This task taps into natural conceptual structures in semantic memory (Deese, 1965; Nelson et al., 2000; De Deyne et al., 2016a). Free associations reflect lexical knowledge acquired through experience and thus can be used to represent the organization of concepts in semantic networks (Siew et al., 2019). As a secondary, exploratory analysis, we also examine sex differences, by collapsing data across the age generation cohorts and dividing the data by sex (male and female).

If *Beauty* and *Wellness* are distinct concepts, we expect distinct and separate “semantic neighbors” surrounding each concept. If these concepts overlap, we expect to find less separated neighborhoods, and that both concepts would be mostly related to terms that are members of the *Beauty* + *Wellness* category. Additionally, based on previous studies on the “aging lexicon” (Wulff et al., 2019), we expected to find increased segregation and decreased connectivity in the semantic networks of older generations cohorts. Finally, if men and women represent differently the concepts *Beauty* and *Wellness*, these differences would be evident in their corresponding semantic networks. For example, do men and women weight physical attributes differently when thinking of beauty or wellness?

## Materials and Methods

### Study Design

Our study has a between-subject age generation cohort design. We collected free association responses to estimate the semantic network of the concepts beauty and wellness and how they relate to each other across the different groups. We used computational linguistic methods (Mikolov et al., 2013) to identify candidate terms (using 100 million words from a decade of published Google News) that relate to the concepts *Beauty* and *Wellness* (top 15 most frequent terms for each concept). To examine the relation of *Beauty* and *Wellness*, we identified terms that related to a category that combined both concepts (*Beauty* + *Wellness*, 15 terms). Such computational methods have been used widely in psycholinguistic research (Mandera et al., 2015, 2017) as a method to identify category related terms. We then collected free-association responses to these 45 terms as well as to the terms *Beauty* and *Wellness* from samples of participants of our targeted four age generations cohorts. Next, we applied a computational approach developed by Kenett et al. (2011) to represent semantic networks derived from these free associations. We estimated a general semantic network of all terms, collapsed across conditions (to address question 1 and 2); the semantic networks for each of the age cohorts (to address question 3); and the semantic network of these terms for men and women, collapsed across all conditions (to address question 4). We then measured different properties of these networks, focusing on the semantic neighbors of the concepts *Beauty* and *Wellness* and their level of integration with each other.

### Participants

Participants were recruited online via Amazon Mechanical Turk (AMT; Buhrmester et al., 2011). We aimed to recruit participants from our four age cohorts, but not to overly constrain random selection of participants. As such, participants were assigned to approximate Generation Z (GenZ; 20–30 years old), Millennials (Mill; 31–40 years old), Generation X (GenX; 41–50 years old), and Baby Boomers (Boomers; 61–70 years old) cohorts. Data were collected in batches of 50 participants at a time and were constrained to specific age groups after a specific age group reached 100 participants. All participants were United States citizens and had English as their native language. To address potential issues with AMT data quality (Chmielewski and Kucker, 2020), we manually scrutinized the raw associative responses data (see below). Exclusion criterions were: (1) Participants who did not respond to more than 10% of the cue words in the free association task were excluded; (2) Participants who generated, on average, a number of associative responses per cue word that was two standard deviations below the group mean were excluded; and (3) Participants who entered non-words as associative responses were excluded. Eight GenZ and three Boomers participants were excluded for generating a low number of associative responses per cue-word (e.g., one associative response). Four additional GenZ participants were excluded because they did not comply with task instructions. Table 1 lists demographic information of the participants in all groups. No statistical differences were found across the cohort groups for years of education or the average number of associations generated by the groups across the different cue words. This study was approved by the University of Pennsylvania Institutional Review Board.

**TABLE 1:** Demographic information on the three groups.

	GenZ	Mill	GenX	Boomers
N	88	118	151	97
%M/%F	63/37	63/37	50/50	38/62
Age	26.5 (2.1)	33.0 (2.2)	46.4 (4.4)	60.1 (5.4)
Education	15 (1.7)	15 (1.9)	15 (2.4)	15 (2.1)
Associations	6.67 (2.66)	7.06 (2.50)	7.45 (2.65)	7.37 (2.16)

*N–number of participants. %M/%/F–percentage of male and female participants. Age–average age in years. Education–average number of education years (standard deviation in parentheses). Associations–averaged number of associations generated across over all cue words.*

### Measures

#### Stimuli Construction

We started with terms that relate to *Beauty* and *Wellness* and terms that bridge the two concepts. To identify such terms, we examined the patterns of natural language use in English (Chowdhury, 2003), using the computational semantic approach, word2vec (Mikolov et al., 2013). Word2vec focuses specifically on word-level textual corpora, trained on a corpus of 100 billion words scraped from Google News over 10 years. The model contained 300-dimensional vectors for 3 million unique words and phrases. Word2vec focuses on the context of individual words, and through a deep neural network predictive modeling approach predicts a target word from a sample of closely co-occurring words (i.e., context words). Previous work reports that this method outperforms traditional computational semantic models, such as latent semantic analysis (Mikolov et al., 2013). We implemented word2vec via the Natural language Toolkit (Edward and Steven, 2002) and the Gensim (Rehurek and Sojka, 2010) python packages. We computed the top 10% (300K) most frequently appearing words and then computed ∼700 word-vectors from this list most similar (cosine similarity) to the word-vector *Beauty*, the word-vector *Wellness*, and the word-vector obtained by adding the word vectors of *Beauty* and *Wellness*. From these lists of terms, we selected 15 terms that appeared in only one list. We did so by selecting terms based on descending frequency of occurrence in the corpus, in relation to each of these vectors. Word2vec vectors are based on 10 years of Google news articles, and it has been argued that it captures mental thought in a broad sense (Mikolov et al., 2013; Mandera et al., 2017). As such, we assume that these terms capture our multidimensional vectors of interest (*Beauty*, *Wellness*, and *Beauty* + *wellness*). Finally, these vector related terms as corpus-based categories offer a benchmark against which to compare to our empirically collected data. Cue words were checked for concreteness using norms of concreteness ratings for 40,000 known English lemmas (Brysbaert et al., 2014). No significant differences were found for the rated concreteness of the words across the three groups (all *p*’s ns).

#### Continuous Free Associations Task

Participants were presented with a cue word and had 1 min to generate as many associative responses as possible they could for that cue word. Participants generated single word free associations to the list of 47 cue words. These 47 cue words consisted of the top 15 words related to *Beauty*, *Wellness*, and *Beauty* + *Wellness*, with the additional concepts *Beauty* and *Wellness* as core words (Appendix Table A1). The Continuous free association task has been shown to be superior to discrete free association tasks in revealing weaker associative relations (Hahn, 2008; Kenett et al., 2011; De Deyne et al., 2013). Continuous free association tasks vary in the amount of time or number of responses required from participants (Kenett et al., 2011). Here, we follow previous studies in having participants generate unrestricted number of associative responses for 1 min per cue word (Kenett et al., 2011; Kenett and Thompson-Schill, 2020). The data were preprocessed to standardize responses and to fix spelling mistakes, e.g., plural responses were converted to singular form, non-words were removed, and capital letters were changed to lower letters. Furthermore, any of the cue words generated as associative responses were also removed.

### Semantic Network Estimation

The various semantic networks were computed and compared using a computational approach developed by Kenett et al. (2011). The core idea of this method is the definition of connections between concepts in the semantic network as an overlap of associative responses generated to these concepts. This notion accords with Collins and Loftus (1975) definition of semantic similarity (Kenett et al., 2011). Currently, growing evidence points to a strong coupling between associative and semantic relations (McRae et al., 2012). Our estimated semantic networks consisted of 47 nodes (cue words from the free association task), that map to the three corpus-based categories.

The associative correlation between any pair of cue words was calculated using Pearson’s correlation. This resulted in a 47 × 47 matrix where each cell denotes the association correlation between node *i* and node *j*. Many edges have small values or weak associations, representing noise in the network. To minimize noise and remove possible spurious associations, we applied a planar maximally filtered graph filter (Tumminello et al., 2005; Kenett et al., 2014; Christensen et al., 2018). This approach retains the same number of edges between the groups and avoids the confound of different network structures arising from different number of edges (van Wijk et al., 2010; Christensen et al., 2018). Thus, networks constructed by this approach can be compared directly because they have the same number of nodes and edges. To examine the structure of the networks, the edges are binarized so that all edges are converted to a uniform weight (i.e., 1). Although the networks could be analyzed using weighted edges (weights equivalent to the similarity strength), this approach potentially adds noise to the structure of the network. Furthermore, Abbott et al. (2015) found that weighted and unweighted semantic network analysis leads to similar results. Thus, the networks are analyzed as unweighted (all weights are treated as equal) and undirected (bidirectional relations between nodes) networks. Estimating semantic networks for different groups (age generation semantic networks) that are comprised from the same nodes (47 cue words) and with an equal number of edges (270 edges) allows comparison between them. Furthermore, the average degree, the average amount of edges per node in all networks, was equal (average of 5.74 edges per node).

Network analyses were performed with the Brain Connectivity Toolbox for Matlab (Rubinov and Sporns, 2010). The clustering coefficient (CC) (measuring network connectivity) and the average shortest path length (ASPL) (measuring global distances) were calculated (Siew et al., 2019). Lastly, the modularity (Q) measure was calculated (Newman, 2006). CC refers to the extent that two neighbors of a node will themselves be neighbors (i.e., a neighbor is a node *i* that is connected through an edge to node *j*), averaged across all nodes in the network. Thus, a higher CC relates to higher overall connectivity in the network. In semantic networks, such connectivity denotes the similarity between concepts. ASPL refers to the average shortest number of steps (i.e., edges) needed to traverse between any pair of nodes, e.g., the higher the ASPL, the more spread out a network is. Previous research at the semantic level have shown that ASPL between concepts in semantic networks corresponds to participants judgments whether two concepts are related to each other (Kenett et al., 2017; Kumar et al., 2020). The network’s CC and ASPL were evaluated qualitatively against the equivalent parameters in a random network with the same number of nodes and edges (CC_rand_ and ASPL_rand_, respectively). Q estimates how a network breaks apart (or partitions) into smaller sub-networks or communities (Newman, 2006; Fortunato, 2010). It measures the extent to which a network has dense connections between nodes within a community and sparse (or few) connections between nodes in different communities. Thus, the higher Q is, the more the network sorts into subcommunities. Such subcommunities can be thought of as subcategories within a semantic memory network. Previous research has shown that modularity in semantic networks is inversely related to a network’s flexibility (Kenett et al., 2016, 2018).

The group-based network analysis computes a single value for each network measure for the different networks. In order to statistically compare the semantic networks across time points and across groups, we applied a bootstrap method (Efron, 1979) to simulate a large distribution of the network measures from the empirical data and compare partial networks for each of the conditions. The bootstrapping procedure involves a random selection of half of the nodes comprising the networks. Partial networks were constructed for each semantic network separately for these selected nodes. This method is known as the without-replacement bootstrap method (Bertail, 1997). Finally, for each partial network, the CC, ASPL, and the Q measures were computed. This procedure was simulated with 1,000 realizations. The difference between the bootstrapped partial networks on each network measure was then tested using either *t*-test (for the sex networks) or one-way ANOVA (for the generation networks) analyses.

To examine differences in the organization of concepts related to *Beauty* and *Wellness* in our networks, we conducted several complementary analyses: First, we qualitatively identified and compared the neighbors (directly connected nodes) of the *Beauty* and *Wellness* nodes for each of the networks. This allows examining key terms associated with each of these concepts across each of the networks. Second, we conducted a community detection analysis to examine how the terms (nodes) in the network cluster into well-defined communities (Hayn-Leichsenring et al., 2020). To do so, we use a data driven approach to determine community assignment of each node in all of the networks (Betzel et al., 2017). We applied a modularity maximization approach to partition a network into communities. This approach uses the Louvain modularity method, a greedy stochastic method (Lancichinetti and Fortunato, 2009). Given the stochasticity of this method, the application of the Louvain modularity method is reiterated 1,000 times (Bassett et al., 2011). To resolve the variability across the 1,000 iterations of the community partitions, a consensus analysis was used to identify the optimal community partition that summarizes the commonalities across the entire distribution of partitions (Lancichinetti and Fortunato, 2012; Betzel et al., 2017). The results of this process are data-driven consensus-based identified communities for each of the networks and community assignment of each of the nodes in the network to a specific community. These data-driven partitions are then compared to our corpus-based defined categories, based on the word2vec analysis.

We conducted two types of analysis on these data driven partitions: First, we computed the Rand similarity index between the corpus-based and each of the data-driven partition separately (Rand, 1971; Traud et al., 2011). The Rand similarity index measures the similarity between two partitions, corresponding to the fraction of node pairs identified the same way by both partitions (either together in both or separate in both partitions). Second, we measured the distribution of the different cue-word categories into the different data-driven identified communities for each network separately. We then computed the standard deviation of these distributions as an indicator for how much they differ (Hayn-Leichsenring et al., 2020). A low SD indicates that the word-cue category is largely distributed across the different communities. A high SD indicates that the word-cue category is clustered in one or a small number of communities. These analyses allow examining how well the corpus-based and data-driven partitions align.

### Procedure

Participants signed a consent form and then completed all tasks using Qualtrics^1^. Participants were instructed to generate, in 1 min, as many different single word associative responses they could think of to a cue word. In each trial, the cue word was presented in the center of the screen with a response box below it, where participants typed their responses. Below the response box appeared a timer, counting down from 60 s. After 60 s elapsed, a new trial immediately began. Cue words were presented randomly and after 25 cue words participants had a short break. Finally, participants were asked a few demographic questions. Participants were asked to self-identify their age (“What is your age?”), sex (“What sex were you assigned at birth?”), and years of education (“How many years of education have you completed?”).

## Results

To address our four research questions, we conducted three analyses: Global network analysis, semantic neighborhood analysis, and semantic communities’ analysis. These analyses were conducted across three different types of networks: A general network (including data from all participants), generation age cohort groups based semantic networks, and sex based semantic networks.

### Global Network Analysis

**FIGURE 1:**
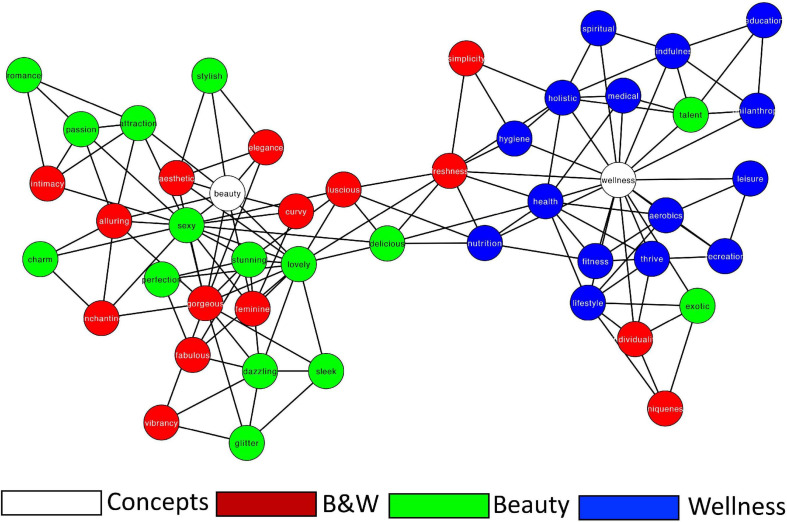
2D visualization of the 47 cue words (nodes) semantic networks. Edges denote symmetrical, binary relations between nodes. Colors represent category membership.

We began by estimating a semantic network based on the entire collected data. Such an estimated general semantic network provides us a baseline for comparison with the different group-based networks we further analyze (sex and generation networks). Next, we computed the network measures of this general network (Table 2). To visualize the networks (Figure 1), we applied the force-directed layout (Fruchterman and Reingold, 1991) of the Cytoscape software (Shannon et al., 2003). In these 2D visualizations, nodes are represented by the respective images and edges between them are represented by lines. Since these networks are undirected and weighted, the edges convey symmetrical (i.e., bidirectional) similarities between two nodes. Visual inspection of the networks shows that the cue words *Beauty* and *Wellness* are separated from each other, each surrounded by cue-words in their corresponding category, while cue-words from the *Beauty* + *Wellness* category are distributed across both “semantic neighborhoods.”

**TABLE 2:** Network measures for the different networks.

	General
CC	0.72
ASPL	2.94
Q	0.47
CC_rand_	0.14
ASPL_rand_	2.33

*CC–clustering coefficient; ASPL–average shortest path length; Q–modularity measure; CCrand–Clustering coefficient of random graph; ASPLrand–average shortest path length of random graph.*

#### Interim Observations

The conceptual space of *Beauty* and *Wellness* segregate into two communities. The terms that linked *Beauty* + *Wellness* based in the word2vec selection criteria do not appear to bridge the concepts. These presumptively combination terms appear to link more closely to the concept of *Beauty*. Finally, terms such as *Talent*, *Exotic*, *Uniqueness*, and *Individuality*, contrary to the word2vec analysis, were more closely related to *Wellness* than to *Beauty*.

#### Age-Generation Cohort-Based Networks

**FIGURE 2:**
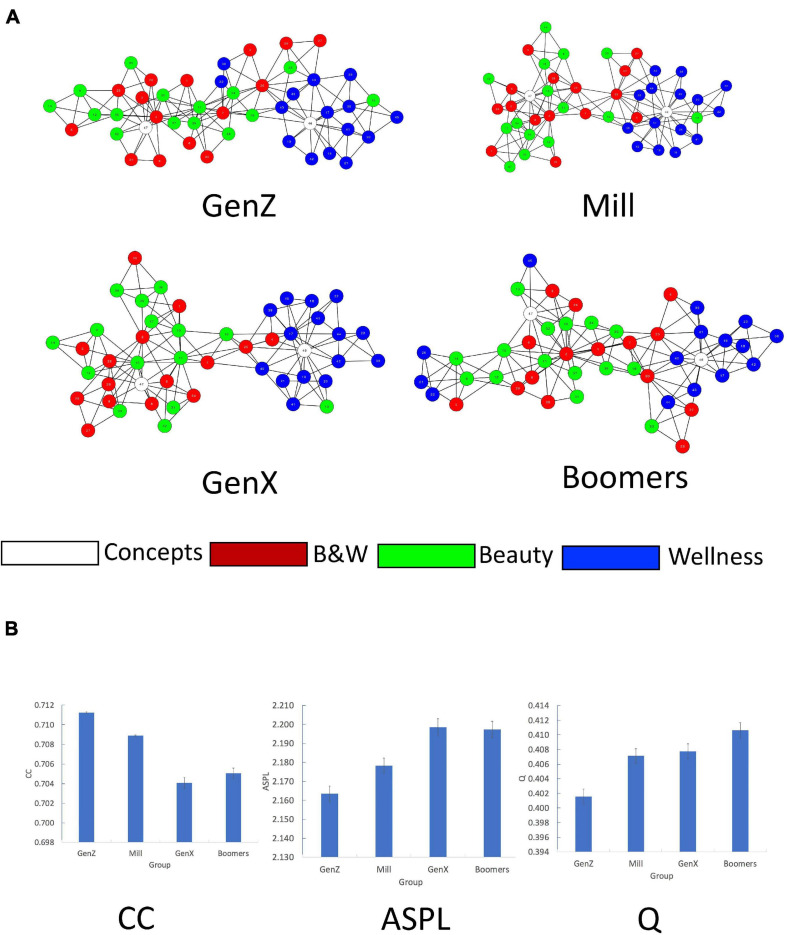
Network analysis for Age Generation. **(A)** 2D visualization of the Age Generation 47 cue words (nodes) semantic networks. Edges denote symmetrical, binary relations between nodes. Colors represent category membership. Node numbers correspond to their word labels that appear in Appendix Table A1. **(B)** Bootstrapping analysis of CC, ASPL, and Q for the Age Generation groups. *X*-axes–Group, *Y* = axes–network measure (CC, ASPL, and Q) values.

Next, we estimated the semantic networks for the GenZ, Mill, GenX, and Boomers generation cohorts. Similarly, to the general network, we computed the network measures (Table 3) and visualized the networks with the Cytoscape software (Figure 2A). The network analysis computes a single value for each network measure for the different networks. To statistically examine the differences in the network measures across the four cohorts, we apply the bootstrapped partial networks analysis (Bertail, 1997; Kenett et al., 2014) to generate a distribution of values for each of the network measures from the empirical data. This method randomly chose half of the nodes comprising the entire network (23 out of 47 nodes). Next, partial networks were constructed for both groups for this subset of nodes. Network measures were computed for each partial network and this procedure was reiterated 1,000 times. This resulted in a sample distribution of 1,000 samples for all measures (CC, ASPL, and Q) for all four networks, which we test via a one-way ANOVA on the group effect of each of the network measures (Figure 2B).

**TABLE 3:** Network measures for the different networks.

	GenZ	Mill	GenX	Boomers
CC	0.72	0.71	0.72	0.71
ASPL	2.86	3.01	2.86	2.95
Q	0.48	0.51	0.51	0.52
CC_rand_	0.08	0.08	0.09	0.09
ASPL_rand_	2.30	2.29	2.28	2.29

*CC–clustering coefficient; ASPL–average shortest path length; Q–modularity measure; CCrand–Clustering coefficient of random graph; ASPLrand–average shortest path length of random graph.*

##### CC

An Age Generation (GenZ, Mill, GenX, and Boomers) one-way ANOVA revealed a significant main effect of Age Generation on CC, *F*(3, 3996) = 39.43, *p* < 0.001, η^2^ = 0.03. *Post-hoc* paired-samples *t*-test analyses reveal that this effect is driven by difference in the change in CC across the Age Generation networks: A significant decrease in CC for the GenZ group compared to the Mill group, *t*(1998) = 3.22, *p* < 0.001, *d* = 0.13; A significant decrease in CC for the Mill group compared to the GenX group, *t*(1998) = 6.38, *p* < 0.001, *d* = 0.29; Finally, no significant differences are found between the CC of the GenX group compared to the Boomers group, *p* > 0.20, *d* = 0.06.

##### ASPL

An Age Generation (GenZ, Mill, GenX, and Boomers) one-way ANOVA revealed a significant main effect of Age Generation on ASPL, *F*(3, 3996) = 15.40, *p* < 0.001, η^2^ = 0.01. *Post-hoc* paired-samples *t*-test analyses reveal that this effect is driven by difference in the change in ASPL across the Age Generation networks: A significant increase in ASPL for the GenZ compared to the Mill group, *t*(1998) = −2.52, *p* < 0.01, *d* = 0.12; A significant increase in ASPL for the Mill group compared to the GenX group, *t*(1998) = −3.37, *p* < 0.001, *d* = 0.16; Finally, no significant differences are found between the ASPL of the GenX group compared to the Boomers group, *p* > 0.85, *d* = 0.01.

##### Q

An Age Generation (GenZ, Mill, GenX, Boomers) one-way ANOVA revealed a significant main effect of Age Generation on Q, *F*(3, 3996) = 13.37, *p* < 0.001, η^2^ = 0.01. *Post-hoc* paired-samples *t*-test analyses reveal that this effect is driven by difference in the change in Q across the Age Generation networks: A significant increase in Q for the GenZ group compared to the Mill group, *t*(1998) = −3.83, *p* < 0.01, *d* = 0.15; No significant difference in Q for the Mill group compared to the GenX group, *p* > 0.66, *d* = 0.03; Finally, a marginal significant difference in Q is found for the GenX group compared to the Boomers group, *t*(1998) = −1.93, *p* < 0.05, *d* = 0.09.

#### Interim Observations

Our findings are similar to previous observations (Dubossarsky et al., 2017; Wulff et al., 2018), demonstrating that older generation cohort groups have more organized, segregated semantic networks (lower CC, higher ASPL and Q). Furthermore, the semantic neighborhoods of *Beauty* and *Wellness* were more segregated from each other in the older generation cohort.

#### Sex-Based Networks

**FIGURE 3:**
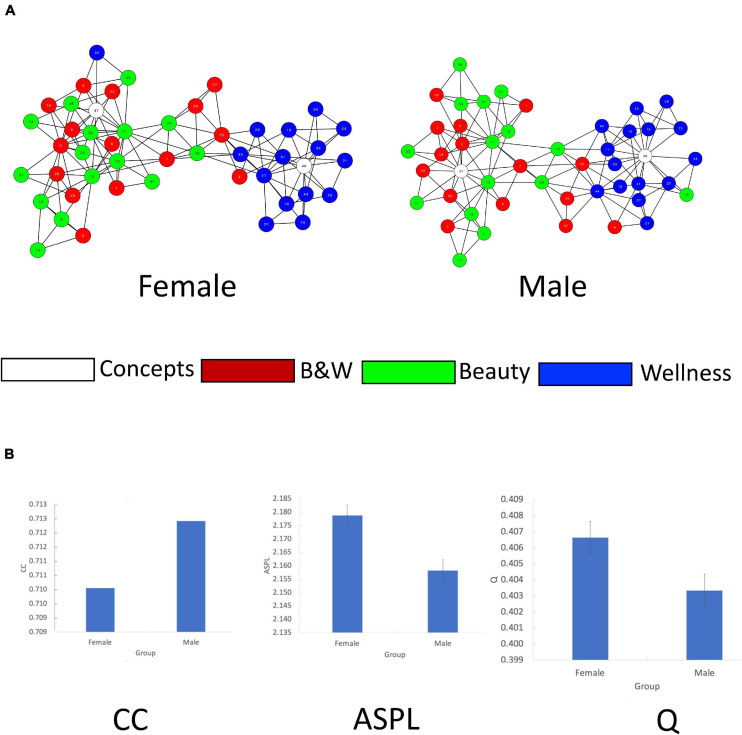
Network analysis for Sex. **(A)** 2D visualization of the Sex 47 cue words (nodes) semantic networks. Edges denote symmetrical, binary relations between nodes. Colors represent category membership. Node numbers correspond to their word labels that appear in Appendix Table A1. **(B)** Bootstrapping analysis of CC, ASPL, and Q for the Sex groups. *X*-axes–Group, *Y* = axes–network measure (CC, ASPL, and Q) values.

Finally, for our exploratory analysis, we estimated the Female and Male semantic networks, collapsing across the different age generation cohorts. We then computed and compared their network measures (Table 4), and similarly visualized the networks using the force-directed layout of the Cytoscape software (Shannon et al., 2003) to plot the graphs (Figure 3A).

**TABLE 4:** Network measures for the different networks.

	Female	Male
CC	0.72	0.71
ASPL	2.95	2.90
Q	0.46	0.51
CC_rand_	0.14	0.13
ASPL_rand_	2.32	2.75

*CC–clustering coefficient; ASPL–average shortest path length; Q–modularity measure; CCrand–Clustering coefficient of random graph; ASPLrand–average shortest path length of random graph.*

To statistically examine possible differences in the network measures across the networks, we similarly apply the bootstrapping analysis approach. We then conduct *t*-test analyses of the sex effect on each of the network measures (CC, ASPL, and Q; Figure 3B). An independent *t*-test analysis on CC revealed that the Female network had a significantly smaller CC (*M* = 0.710, SD = 0.02) than the Male network (*M* = 0.712, SD = 0.02), *t*(1998) = −3.55, *p* < 0.001, *d* = 0.10. An independent *t*-test analysis on ASPL revealed that the Female network had a significantly larger ASPL (*M* = 2.179, SD = 0.12) than the Male network (*M* = 2.158, SD = 0.13), *t*(1998) = 3.73, *p* < 0.001, *d* = 0.17. An independent *t*-test analysis on Q revealed that the Female network had a significantly larger Q (*M* = 0.407, SD = 0.032) than the Male network (*M* = 0.403, SD = 0.032), *t*(1998) = 2.31, *p* = 0.021, *d* = 0.13.

#### Interim Observations

Overall, these findings reveal that the Male semantic network is less structured than that of the Female semantic network (higher CC, lower ASPL and Q). The communities around *Beauty* and *Wellness* are more segregated for women than for men. As representative of qualitative differences observed, women relate *Education* to *Beauty*, while men relate *Talent* to *Wellness*.

### Semantic Neighborhood Analysis

**FIGURE 4:**
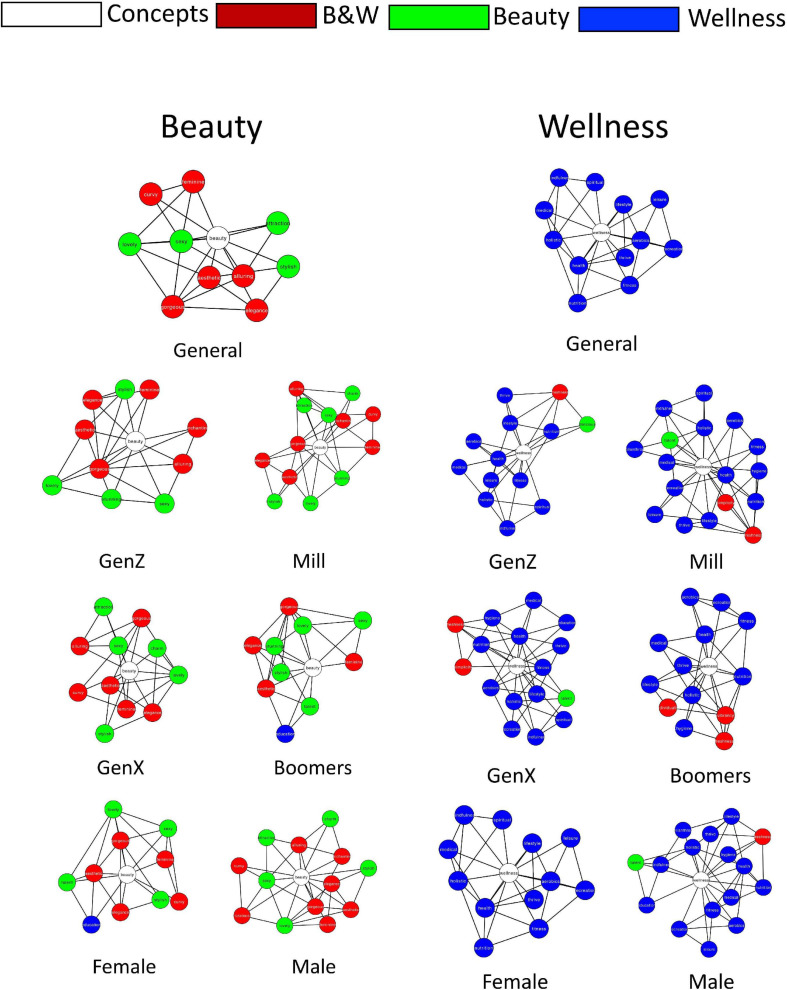
Semantic neighbors of *Beauty* and *Wellness* across the seven estimated semantic networks.

Next, we qualitatively identified and compared the direct neighbors (directly connected nodes) of the cue words *Beauty* and *Wellness* across the seven networks that we examined (General, Female, Male, GenZ, Mill, GenX, and Boomers; Figure 4) to identify similarities and differences in their neighbors. Specifically, for *Beauty*, the terms *Elegance*, *Feminine*, *Gorgeous*, *Lovely*, *Sexy*, and *Stylish* were direct neighbors across all networks. For *Wellness*, the terms *Aerobics*, *Fitness*, *Health*, *Holistic*, *Lifestyle*, *Medical*, *Nutrition*, and *Thrive* were direct neighbors across all networks. Furthermore, a few terms originally assigned to *Beauty* or *Wellness* ended up being direct neighbors of the other concept: The term *Education* (originally assigned as a *Wellness* category) was a direct neighbor of *Beauty* (in the Female and Baby Boomers networks); whereas the terms *Delicious*, *Exotic*, and *Talent* (originally assigned as *Beauty* categories) were direct neighbors in some of the *Wellness* networks (Supplementary Tables 1, 2). Finally, we examined the categories that comprise the direct neighbors of *Beauty* and *Wellness* (Supplementary Tables 1, 2). We find that the direct neighbors of *Beauty* came from the *Beauty* + *Wellness* category (8/16 overall neighbors) and the *Beauty* category (7/16 overall neighbors). In contrast, the direct neighbors of *Wellness* are comprised from the entire *Wellness* category (15/22) with only three direct neighbors from the *Beauty* + *Wellness* category (3/22). This finding indicates that the *Beauty* + *Wellness* category as derived from the word2vec analysis is more closely related to the concept of *Beauty*, and that terms in the *Wellness* category derived similarly are more directly related to the concept of *Wellness*.

### Semantic Community Analysis

**FIGURE 5:**
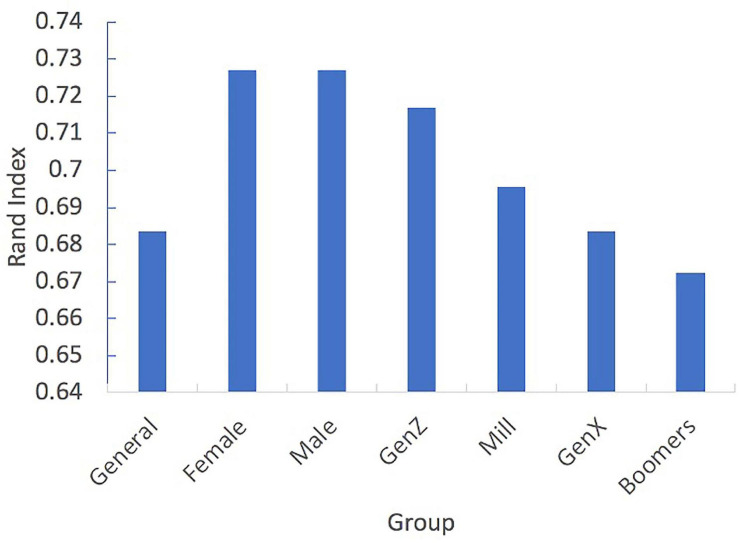
Rand similarity index between corpus-based partition (cue word categories) and each of the seven data-driven consensus partitions for each of the networks.

Finally, we conducted data-driven community detection analysis to compute the consensus partition for each of the seven networks. We computed the Rand similarity index to measure the similarity of each of the data-driven partitions and the corpus-based partition (Figure 5). The higher the Rand similarity index is, the more similar the data-driven partition is to the corpus-based partition. This analysis revealed that while the General network was highly similar to the corpus-based partition (Rand index = 0.68), the sex-based networks were the most similar to the corpus-based partition (Rand index = 0.73 for both networks). Furthermore, this analysis reveals that as the age of the cohort increased, the overall similarity between the consensus partition to the corpus-based partition decreased (from a Rand index = 0.72 for GenZ to a Rand index = 0.67 for Baby Boomers). This finding is in line with our findings on the varying network properties over the different generations: In older generation cohorts, networks are more segregated.

Next, we examined how well the corpus-based categories map on to the data-driven identified communities in each of the networks we analyze. For each network, we measured how many category members of a specific category map on to the different communities identified in that specific network. Then we computed the standard deviation of the distribution of the different category words across these communities (Table 5). A low SD score indicates that a category is equally distributed across the communities while a high SD score indicates that a category largely maps onto one or a few number of categories (Hayn-Leichsenring et al., 2020). This analysis reveals that in all networks the cue words *Beauty* and *Wellness* always map onto different communities (leading to a SD of 0). Furthermore, in all networks, words related to the *Wellness* category are the most dispersed across communities (having the lowest SD). Finally, across all networks, the *Beauty* + *Wellness* category has the highest SD, indicating that cue words in this category largely map to a small number of clusters (2–3), with a majority of the cue words in one community. This finding may be related to our findings that most cue words in this category relate to the concept of *Beauty*.

**TABLE 5:** Standard deviations (SD) of the distribution of the different category words into the identified communities in the different semantic networks.

	General	Female	Male	GenZ	Mill	GenX	Boomers
Beauty	2.92	3.40	2.83	2.75	2.92	2.92	2.74
Wellness	1.87	2.22	1.87	2.12	1.87	1.87	2.45
Beauty + Wellness	7.78	6.93	9.19	7.78	4.95	3.54	4.27
Concepts	0	0	0	0	0	0	0

*Lower SD represents a more equal distribution.*

## Discussion

People are pre-occupied by notions of beauty and wellness (Grammer et al., 2003; Koskinen et al., 2017). As a concrete representation of this pre-occupation, the world of commerce links beauty to wellness in the sales of their products and services. The Global Wellness Institute reports that the beauty is the largest commercial sector at a billion dollars in an overall 4.2 trillion dollar wellness market (Global Wellness Institute, 2017). In this study, we asked: How do people think about beauty? About wellness? Are these concepts linked and do they vary by aged and sex?

To address these questions, we applied computational network science methods to analyze free-association responses generated to terms related to beauty and wellness, collected from a large sample of participants that varied in sex and age (Kenett et al., 2011; Siew et al., 2019). This approach allowed us to ask these questions across different resolutions: A general network, comprised from the entire data, served as a benchmark; age generation cohort-based networks, to compare differences in people of different ages; and sex-based networks, compared differences between men and women.

Our general network revealed two distinct semantic neighborhoods, surrounding each of the terms *Beauty* and *Wellness* separately. Thus, these two concepts are conceptually separable, with related terms that link them. Our work is in line with previous lexical based analysis of aesthetic concepts (Kuehnast et al., 2014; Hosoya et al., 2017; Menninghaus et al., 2019). For example, Menninghaus et al. (2019) found that elegance, grace, and sexy were terms that largely overlapped with the concept beauty, but also differed from each other with regard to their unique meanings. Our approach extends their study by examining a broader range of terms related to beauty (see Sabb et al., 2008; Sabb et al., 2009 for a similar arguement).

We found that *Beauty* was closely related to *Elegance*, *Feminine*, *Gorgeous*, *Lovely*, *Sexy*, and *Stylish*. These findings suggest that some associations to *Beauty* are consistent across sex and age and incorporate physical and shared cultural notions. For *Wellness*, the terms *Aerobics*, *Fitness*, *Health*, *Holistic*, *Lifestyle*, *Medical*, *Nutrition*, and *Thrive* were also linked directly in all networks. These associations suggest that wellness is associated with active practices that promote health and thriving; the latter implying a more abstract sense of fulfilling our potential.

Our cohort network analysis revealed that older generations had more organized and segregated semantic networks (lower CC; higher ASPL and Q). These findings are similar to previous studies on the structure of the mental lexicon across cohorts (Zortea et al., 2014; Dubossarsky et al., 2017; Wulff et al., 2018, 2019; Cosgrove et al., 2021). Furthermore, we found two effects of the age cohort on the semantic neighborhood of *Beauty* and *Wellness*: First, for older cohorts, the semantic neighborhoods of *Beauty* and *Wellness* were more segregated from each other. Second, the semantic network of the Baby Boomers was the most segregated, precipitating into many sub-communities, potentially indicating narrower and more nuanced dissection of these concepts. Two possible hypotheses can account for these cohort effects: The first is based on more lived experience–older adults accumulate more knowledge, which leads to heightened segregation in their semantic memory network. The second is based on socio-cultural dynamics–older adults are increasingly aware of stereotypical models of beauty and wellness related to young, healthy bodies, awareness that develops their notion of these concepts, leading to heightened segregation in their semantic memory network. Our findings cannot distinguish between these two competing hypotheses, which requires additional, follow up research to be examined directly.

Comparing the sex-based semantic network, we found differences in how females and males think of these concepts. The Female semantic network was more organized with the concepts *Beauty* and *Wellness* being more distinct, while the Male semantic network had two semantic neighborhoods, for *Beauty* and *Wellness*. Both the Female and Male semantic networks had semantic neighborhoods (“communities” of terms) around the concepts of *Beauty* and *Wellness*. The Female semantic network, however, had a third intermediate and smaller community between the neighborhoods of *Beauty* and *Wellness*, serving as a “bridge” community in between the *Beauty* and *Wellness* semantic communities (Figure 3). This “bridge” community included terms that focus on an appearance and individuality (*Exotic*, *Luscious*, *Delicious*, *Uniqueness*, and *Individuality*).

Next, we examined which terms comprise the semantic neighborhoods of *Beauty* and *Wellness* across the various networks (General, Sex-based, Generation-based). This analysis allows us to identify the terms relevant to these hard-to-define concepts and examine how they vary in relation to age and sex (Supplementary Tables 1, 2). Overall, we found similarities and differences for these semantic neighborhoods across the various networks. In comparing the male and female networks, the male *Beauty* semantic neighborhood had more terms from the *Beauty* + *Wellness* category that related to physical appearances (e.g., *Alluring*, *Attraction*). As for the *Wellness*, the male semantic neighborhood was also larger, and included more general terms (e.g., *Education*, *Hygiene*).

The semantic neighborhood of *Beauty* for the Millennials and Generation X groups had unique terms related to appearance (e.g., *Attraction*, *Curvy*), compared to the two other groups. Furthermore, the Baby Boomers cohort was the only group that had *Education* as part of the semantic neighborhood of *Beauty*. As for age generation differences in the semantic neighborhood of *Wellness*, the semantic neighborhoods of *Wellness* across the age generation cohorts were mostly similar, but also had unique terms for the Generation Z (*Delicious*), Millennials (*Philanthropy*), Generation X (*Education*, *Spiritual*), and Baby Boomers (*Individuality*, *Vibrancy*). One could speculate that millennials are attuned to social and communal wellbeing and older people are more concerned with their personal wellbeing.

We demonstrate that methods from network science can be applied to investigate abstract concepts such as *Beauty* and *Wellness*. What people mean by these terms can be highly variable and yet they seem to have a shared understanding. Our method of analysis offers a window into the conceptual space occupied by these terms common to how people think and also to how they differ across demographic variables. Critically, our method provides a quantitative, empirical driven approach to analytical philosophical investigations of conceptual analysis (Wittgenstein, 1953; Rosch, 1975; Lakoff and Johnson, 1980; Ramsey, 1992). An underlying assumption in conceptual analysis—the analysis of the meaning of concepts conducted by philosophical inquiry—is that there is a cognitive representation of knowledge, as proposed by Collins and Loftus (1975). One way that conceptual analysis is conducted is by philosophers proposing and rejecting definitions for a concept, based on intuition and logicism (Ramsey, 1992). Our approach, in contrast, combines empirical data collection with computational representation of semantic memory as networks and uses the semantic neighborhood of a concept as a way to define it. This offers a quantitative approach that can facilitate such conceptual analysis. In addition, our approach resonates with the distributional hypothesis, that states that a concept is “characterized by the company that it keeps” (Harris, 1970).

Side stepping philosophical and conceptual challenges of defining such terms (Corbin and Pangrazi, 2001; Scruton, 2011; Redies, 2014; Menninghaus et al., 2019), we approach this challenge from an empirical, data-driven endeavor. Our approach focuses on the semantic neighborhoods of *Beauty* and *Wellness* and allows us to examine how these neighborhoods relate to each other. Our approach informs us about the stability and variability in conceptualizing beauty and wellness across age and sex. Importantly, it affirms modern characterization of both of these concepts to reflect physical, mental, and cultural attributes. Indeed, our results indicate that beauty is about being elegant and sexy at the same time; wellness is about being healthy and spiritual at the same time. Our findings indicate that a core meaning of these two concepts, operationalized by their semantic neighborhood, is stable across age and sex and age.

The cultural salience of these concepts is reflected in the fact that beauty and wellness industries are booming. People avidly pursue and consume beauty and health related products (Koskinen et al., 2017). Visiting fitness centers and beauty salons, taking vitamins, reading self-help books and wellness blogs, going on activity holidays or quieting down at silent retreats are all examples of wellness practices today, that are practiced across all ages (Crawford, 2006; Koskinen et al., 2017).

Our findings convey insights about beauty and wellness that can be used in an applied manner. Given the current massive beauty and wellness industries (Global Wellness Institute, 2017), there is great commercial interest in how these concepts relate for different sex and age potential consumers (Chen et al., 2015; Ostapenko, 2015; Valentine, 2016; Khan et al., 2017; Lee et al., 2019). Our findings regarding terms that stay stable or change as direct neighbors of the concepts beauty and wellness may be used to identify the associated relevance of terms to specific populations. A scrutiny of Figures 1–3 and Supplementary Tables 1, 2 offers a rich source of how conceptualization of these terms vary by demographics. For example, in the Female semantic network, the neighborhoods of *Beauty* and *Wellness* are connected via an intermediate subcommunity of terms that relate to physical attributes and pleasure from food. Women relate *Education* to *Beauty*, while men relate *Talent* to *Wellness*.

Our findings may also impact philosophical aesthetical conceptualization of the notions of beauty and wellness (Zangwill, 2005, 2018; Scruton, 2011, 2018; Sartwell, 2017). For example, perhaps the main philosophical debate on beauty is whether beauty is subjective, e.g., “in the eye of the beholder,” or whether it is an objective feature of beautiful things (Sartwell, 2017). Our findings of terms that are semantic neighbors of the term *Beauty* and are stable across all network comparisons we conducted may contribute to this philosophical debate. These stable semantic neighbors may indicate an objective aspect of beauty, that is shared across people that vary in age and sex. Thus, the application of network science methodologies on empirical human data may inform philosophical discussions, discussions that go all the way back to ancient Greece (Sartwell, 2017).

Our study has several limitations. One limitation is that we cannot distinguish whether our age generation effect is due to a cohort effect or reflects changes across the lifespan. This limitation applies to other similar “aging” studies (Dubossarsky et al., 2017; Wulff et al., 2018; Cosgrove et al., 2021). Longitudinal research is needed to examine effects of aging on the evolution of the concepts of beauty and wellness (Costanza et al., 2012, 2017; Costanza and Finkelstein, 2015; Rudolph et al., 2020a). While cross-sectional age generation cohort analysis is common in aging studies (Costanza et al., 2012), longitudinal and time varying methods to study individual variation in age, compared to aggregated groups, should be applied in such follow up studies to our current research (Costanza et al., 2017). Such an individual-based research is crucial given that age-generation classification varies across cultures and individuals’ self-identity.

A second limitation is that our semantic networks were small (47 nodes) and comprised solely from terms that were identified as strongly related to either the terms *Beauty*, *Wellness*, or *Beauty* + *Wellness*. Perhaps the stability we find in the semantic neighborhoods of *Beauty* and *Wellness* arise from an “under-sampling” of the mental lexicon. In addition, while we find significant differences in the network measures across our different analyses, the effect sizes are extremely small. Such small effect sizes may be due to the small size of these networks. Additional study with a larger number of terms and categories is required to replicate and generalize our findings.

A third limitation is that our analysis is conducted at the group level, aggregating over participants for the different conditions we examined. Thus, a similar analysis as conducted in our study at the individual level would be helpful. While currently still a challenge, a few methods have been proposed to estimate individual-based semantic networks (Morais et al., 2013; Zemla et al., 2016; Benedek et al., 2017; He et al., 2020; Wulff et al., 2021). Thus, follow up research is needed to estimate individual-based semantic networks related to the concepts of beauty and wellness, to replicate and extend our current findings.

A fourth limitation is related to how we identified and selected the different terms we use for our semantic network analysis, via word2vec analysis of the Google news corpus. Research has indicated that performance of semantic space models such as word2vec strongly depends on the choice and scope of the text corpus used, which can become the determining factor in how well the model captures human performance (Recchia and Jones, 2009). In regard to our study, the specific newspaper sources and articles that are analyzed from the Google news corpus to extract the word vectors related to beauty and wellness may be biased. Such bias may be related to narrow scope of meanings related to these terms or biased by specific age of the authors. Thus, additional research is needed to replicate and extend our findings by using semantic spaces based on other textual corpora which will allow verifying and generalizing our findings.

A fifth limitation relates to our collected sample. In our study, we only focused on native English speakers living in the United States. Thus, there are certainly important linguistic and cultural differences related to the perception and comprehension of *Beauty* and *Wellness* across different languages and cultures. For example, Jackson et al. (2019) conducted a large scale computational linguistic analysis of emotion words across 174 languages. The authors found both language and cultural differences but also potentially universal structure in emotion linguistic spaces (Jackson et al., 2019). The work of Jackson et al. (2019) provides a promising future direction for our study, potentially conducting a similar large scale linguistic analysis to identify Beauty and Wellness universal terms and then replicate our empirical semantic network analysis approach.

Finally, our sex-based semantic network analysis was exploratory. While our analysis revealed interesting, preliminary results on differences in how men and women represent the concepts of beauty and wellness, we did not design the study to match the number of men and women across the four age generation cohorts. Thus, additional research is needed to examine such sex-based differences, which are hypothesis driven and matched across the sample of men and women.

A key aspect of our semantic neighborhood and semantic community analyses is based on our corpus-based categories and terms, derived from word2vec (Chowdhury, 2003; Mikolov et al., 2013). While we find that *Wellness* related terms comprise a cohesive semantic neighborhood surrounding the term *Wellness*, the *Beauty* + *Wellness* terms mostly related to *Beauty*. In addition, some terms that were identified via word2vec as related to the *Beauty/Wellness* categories were in the semantic neighborhood of the *Wellness/Beauty* categories. These findings also further illustrate the challenge in using corpus-based approaches (e.g., word2vec) to capture mental thought (De Deyne et al., 2016b; Kenett et al., 2017; Hayn-Leichsenring et al., 2020; Kumar et al., 2020). Thus, our findings relate to an open debate in computational semantics and suggest limitations of corpus-based methods such as word2vec to predict human behavior (Griffiths et al., 2007; Hutchison et al., 2008; Mandera et al., 2015, 2017; Vankrunkelsven et al., 2018; Kenett, 2019; Kumar, 2021).

In conclusion, we applied computational network science methods to study the semantic structure of beauty and wellness, their relation to each other and how they differ across age and sex. Our approach offers an empirical way to clarify the contribution of knowledge in aesthetic experiences in so far as language rather than perception is being used to probe how people think about beauty and wellness (Chatterjee and Vartanian, 2014, 2016). We found that the semantic neighborhoods of beauty and wellness are largely stable across different age and sex. However, we also find unique differences across these comparisons and find generation cohort effects, showing how nuanced associations of these concepts vary by age. Mapping the space of concepts that characterize beauty, wellness, and their connection advances our understanding of these socially salient concepts.

## Data Availability Statement

The original contributions presented in the study are included in the Supplementary Materials, further inquiries can be directed to the corresponding author.

## Ethics Statement

The studies involving human participants were reviewed and approved by University of Pennsylvania Institutional Review Board. The patients/participants provided their written informed consent to participate in this study.

## Author Contributions

All authors conceived and planned the study and wrote the manuscript together. AC provided the theory for the study. YK and LU provided the computational methods to run and analyze the study. YK collected and analyzed the data.

## Conflict of Interest

The authors declare that the research was conducted in the absence of any commercial or financial relationships that could be construed as a potential conflict of interest.

## Publisher’s Note

All claims expressed in this article are solely those of the authors and do not necessarily represent those of their affiliated organizations, or those of the publisher, the editors and the reviewers. Any product that may be evaluated in this article, or claim that may be made by its manufacturer, is not guaranteed or endorsed by the publisher.

## Footnotes

1
www.qualtrics.com

## Appendix

**APPENDIX 1:** Cue-words used in the continuous free association task.

Category	Stimuli
Beauty	Attractive (12); Charm (31); Dazzling (35); Delicious (16); Exotic (33); Glitter (36); Lovely (11); Passion (9); Perfection (37); Romance (14); Sexy (15); Sleek (34); Stunning (10); Stylish (32); Talent (13).
Wellness	Aerobics (42); Education (45); Fitness (19); Health (17); Holistic (41); Hygiene (39); Leisure (38); Lifestyle (44); Medical (18); Mindfulness (20); Nutrition (40); Philanthropy (22); Recreation (23); Spiritual (21); Thrive (43).
Beauty + Wellness	Aesthetic (3); Alluring (26); Curvy (8); Elegance (24); Enchanting (28); Fabulous (1); Feminine (6); Freshness (25); Gorgeous (2); Individuality (27); Intimacy (5); Luscious (7); Simplicity (4); Uniqueness (29); Vibrancy (30).
Concepts	Wellness (46); Beauty (47)

*Numbers (in parentheses) correspond to node labels in Figures 2, 3.*
